# Diagnostic efficacy of Light-Emitting Diode (LED) Fluorescence based Microscope for the diagnosis of Tuberculous lymphadenitis

**DOI:** 10.1371/journal.pone.0255146

**Published:** 2021-07-29

**Authors:** Gebeyehu Assefa, Kassu Desta, Shambel Araya, Selfu Girma, Adane Mihret, Tsegaye Hailu, Abay Atnafu, Nigatu Endalafer, Adugna Abera, Shiferaw Bekele, Leila Birhanu, Getu Diriba, Yordanos Mengistu, Biniyam Dagne, Kidist Bobosha, Abraham Aseffa

**Affiliations:** 1 Armauer Hansen Research Institute, Addis Ababa, Ethiopia; 2 Department of Medical Laboratory Sciences, Addis Ababa University, Addis Ababa, Ethiopia; 3 Ethiopian Public Health Institute, Addis Ababa, Ethiopia; 4 St. Peter Specialized Hospital, Addis Ababa, Ethiopia; Institute of Medical Sciences, Banaras Hindu University, INDIA

## Abstract

**Background:**

The comparatively straightforward and cheaper light-emitting diode fluorescent microscope (LEDFM) was suggested by WHO to replace conventional microscope in tuberculosis (TB) laboratories. However, the comparable efficacy of each of those techniques differs from laboratory to laboratory. We investigated the efficacy of LEDFM for the diagnosis of tuberculous lymphadenitis (TBLN) patients.

**Methods:**

A cross-sectional study was conducted on 211 samples from clinically suspected tuberculous lymphadenitis patients. Three smears were prepared from FNA on microscope slides for cytomorphology study, Auramine O (AO), and for Ziehl-Neelsen (ZN) staining. The left-over samples were inoculated onto Lowenstein-Jensen (LJ) media. Statistical analysis was done using STATA version 11. The sensitivity, specificity, positive and negative predictive values were calculated by considering the culture results as the gold standard using a 95% confidence interval.

**Results:**

Among 211 samples 49.7% (105) were positive by cytomorphology, 32.7% (69) by LEDFM, 23.69% (50) by LJ culture, and 13.7% (29) by ZN. Compared to the gold standard sensitivity of ZN, LEDFM, and cytomorphology were 30% [95% CI: 17.9–44.6], 66% [95% CI: 51.2–78.8] 78% [95% CI: 64–88.5], respectively. The specificity of ZN, LEDFM, and cytomorphology was 91.3% [95% CI: 85.8–95.2], 77.6% [95% CI: 70.4–83.8], 58.8% [95% CI: 50.7–66.5], respectively.

**Conclusion:**

LED fluorescence microscopy gives a legitimate option in contrast to conventional ZN techniques in terms of its higher sensitivity, a bit lower specificity, time-saving, and minimal effort.

## Background

*Mycobacterium tuberculosis* (MTB) a causative agent of tuberculosis (TB) affects the lungs in most cases, but it may also affect other areas of our bodies [1, 2]. TB remains a significant global health problem, accounting for one of the world’s top 10 causes of death. According to WHO, ten million people develop tuberculosis by 2020, with 1.2 million dead from the disease [3].

Extra-pulmonary tuberculosis (EPTB) accounts for 16% of the 7.1 million incident cases of TB that were notified in 2019 worldwide [4]. Even though tuberculous lymphadenitis (TBLN) accounts for the majority of EPTB cases, and also contributes to the burden of diseases, it does not receive specific attention in international control strategies [5]. In developing countries with a high prevalence of tuberculosis, TBLN is one of the most common causes of lymphadenopathy, accounting for (30–52%) [6–10].

Ethiopia is among the 48 high TB burden countries in the world. The proportion of EPTB in the country is very high [11] accounting for 36.6% of all TB cases. Among all EPTB cases, TBLN is dominant comprising 80% of the cases [12, 13]. The identification of infectious cases is a crucial step for TB control programs worldwide. To improve the control of tuberculosis, TB control programs must have access to rapid and accurate laboratory diagnosis. Considering this programming achievement, new tools have been developed in the last decades to improve the laboratory diagnosis of TB [14]. Culture takes 3–8 weeks to demonstrate the growth of the organism [12]. This delay, in turn, leads to unnecessary suffering for the patients. Polymerase Chain Reaction (PCR) is a sensitive and specific method for the diagnosis of EPTB but it is unaffordable to be used for routine diagnostic purposes in most of the health stations in Ethiopia. Ziehl-Neelsen (ZN) staining using Fine Needle Aspirate (FNA) has low sensitivity [15].

FNA cytology may be a good alternative to diagnose TBLN since obtaining an FNA sample causes less physical and psychological discomfort to the patient [12]. In Ethiopia, some diagnostic methods of EPTB detection, i.e., Lowenstein-Jensen (LJ) culture in regional laboratories, cytomorphological examination, and ZN staining are being exercised more frequently. The use of Light Emitting Diode Fluorescence Microscope (LEDFM) has also been introduced currently by the Federal Ministry of Health to regions with high TB burden in Ethiopia. Even though, there are a couple of works performed at specific regions of the country to evaluate the overall efficacy of LEDFM for TBLN diagnosis using FNA samples [16, 17], well representative works are required to show the performance of the microscope concretely [16].

## Material and methods

### Study area, period, and setting

This study was conducted in All African Leprosy and Tuberculosis Rehabilitation and Training Center (ALERT), Armauer Hansen Research Institute (AHRI), and St. Peter TB Specialized Referral Hospitals from January to April 2020 in Addis Ababa, Ethiopia. ALERT and St. Peter TB Specialized Hospital have 25–45 TB lymphadenitis suspected cases per week. The ALERT Hospital pathology unit is linked with Armauer Hansen Research Institute (AHRI) Laboratory and gives service for both patients’ diagnosis and research activities.

### Study design

A cross-sectional study was conducted on clinically suspected TBLN cases (i.e., enlarged lymph node, fistula, cheloid, draining sinus) to determine the diagnostic potential of LEDFM. All TBLN suspected patients visiting the study areas in the given period participated in this study.

### Sampling procedure

Consecutive sampling techniques were employed to include the study participants according to Buderer’s formula to determine the sample size at the required absolute precision level for sensitivity and specificity. Standard procedure was followed by an experienced pathologist to collect FNA specimens.

### Fine needle aspiration (FNA)

A sterile 21-gauge needle was used to collect FNA samples from enlarged nodes, the overlying skin was cleaned with 70% alcohol. The enlarged node was fixed and maintained in a stable position by the left hand of the attending Pathologist. Then the node was entered with a negative pressure applied to the syringe. Multiple (average of six) in and out passes were made by the needle without exiting the node to collect approximately 50–60 microliters of aspirate. After removing the needle, a drop of the collected FNA sample was placed on a clean slide. Three smears were prepared for Cyto-morphological examination, AO staining, and the other for ZN staining. The leftover sample was transferred into Nunc Cryo Tubes containing 1 ml of sterilized normal saline to be used for culture on LJ medium. The smears were air-dried on a rack for auramine O, and ZN staining smears were heat-fixed after air drying [18, 19]. FNA samples collected from St. Peter Hospital were placed on an icebox and transported to AHRI Laboratory for processing as soon as possible. After collection, all of the samples were processed as soon as they arrived at AHRI.

## Laboratory methods

### Auramine O staining

Following air drying smears were covered with 1% auramine O solution for 15 minutes then rinsed with clean running tap water and decolorized with a 1% acid-alcohol for 2 minutes. Again, distilled water was used to wash the smear. Finally; counterstained with 0.5% potassium permanganate for 2–4 minutes, then rinsed with sterile water and allowed to air dry and smears were examined under the LED fluorescence microscope (Primo Star iLED, Carl Zeiss, Gottingen, Germany) at 400X magnification (455nm) [20]. The test result was interpreted as indicated Table 1 in S1 File [21].

### Ziehl-Neelsen staining

Carbol fuchsin stain was applied to cover the entire slide and slowly heated using a Bunsen burner until steam arose and kept for 5 minutes. After this procedure, the slides were rinsed with distilled water and flooded with 3% acid alcohol, and allowed to decolorize for 3 minutes. In the end, the slides were thoroughly rinsed with water and counterstained for 1 minute with 0.1 percent methylene blue. Finally, the washed and dried smears were examined under the bright field microscope (Primo Star iLED, Carl Zeiss, Gottingen, Germany) at 1000X magnification and interpreted as indicated Table 2 in S1 File [21].

### Cytomorphological staining

Air-dried smears were fixed immediately with alcohol and stained with Giemsa stain. Cytological analysis was performed by a senior Pathologist. The standard cytological criteria for diagnosing TBLN are granuloma of epithelioid cells with or without multinucleated giant cells and caseation necrosis [13].

### Culture technique

**Fig 1:**
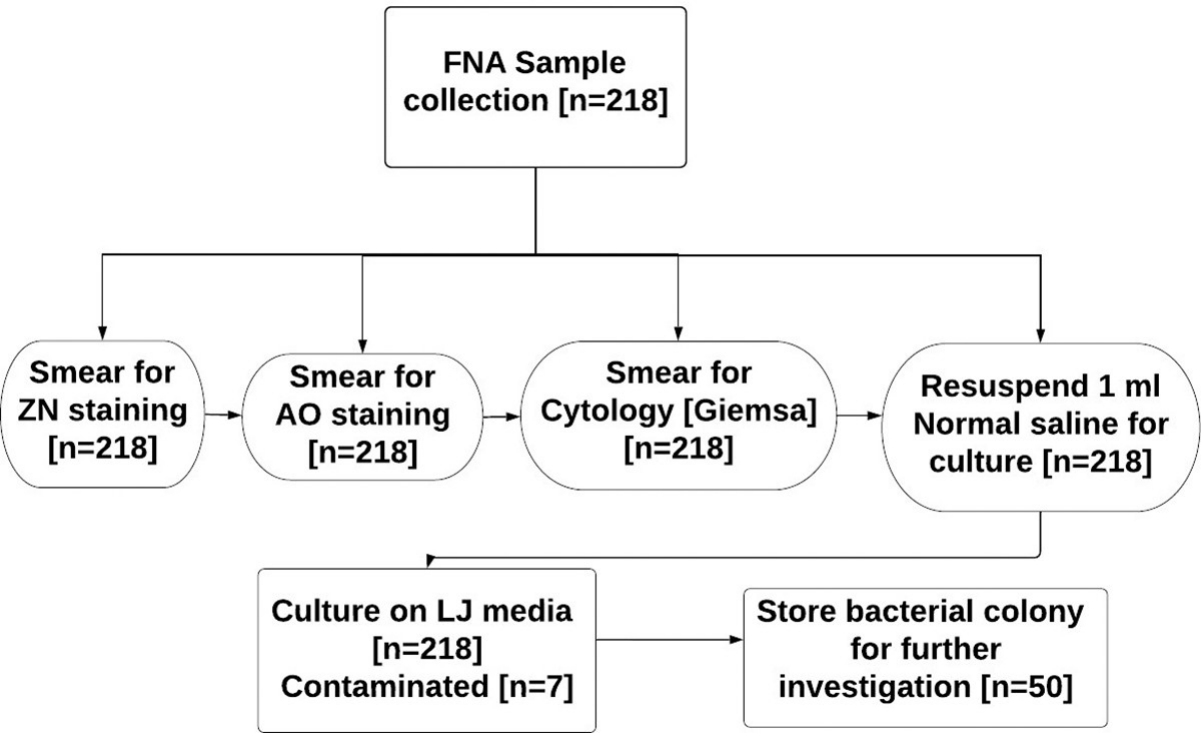
Flow chart of the study procedure.

From the FNA leftover material, culture was performed according to standard procedure [22] on egg-based Lowenstein-Jensen-glycerol media and incubated at 37°C for at least 8 weeks, the culture was observed in a weekly manner to see if there are any mycobacterial colonies. The physical appearance of the media like changing in color from light-green to blue, cottony growth, liquefaction, and breaking of the entire media were considered as contaminated culture. All positive LJ culture which had a buff, rough raised, dry, wrinkled surface, cream-colored, non-pigmented colonies were confirmed for acid fastness by Ziehl-Neelsen staining. No growth over the entire LJ media within 8 weeks was recorded as culture negative. Any single colony on the media was counted as positive for LJ culture (**Fig 1**). The culture result was registered according to the standard reporting scheme as indicated in Table 3 in S1 File [23].

### Data analysis

Statistical analysis was done using STATA 15 (Stata Corporation, College Station, TX). The socio-demographic, physical, and clinical examination data, laboratory results, sensitivity, specificity, positive and negative predictive values including their 95% confidence intervals (CI) were calculated by using the culture results as the gold standard. A Chi-square test was used to assess whether a difference between values obtained is significant. The statistical differences in the tests were considered significant if the two-sided P-value was <0.05.

### Ethical consideration

The study was commenced after obtaining ethical clearance from Addis Ababa University College of Health Science Department of Medical Laboratory Science (DRERC) and the ALERT AHRI ethical review committee. Support letters were obtained from the Department of Research and Ethics Review Committee of Addis Ababa University. Study subjects were informed about the purpose of the research and asked to participate in the study by offering their personal information and FNA specimen. Written informed consent was obtained from all adult participants and parents or guardians in case of children.

## Results

Two hundred and eighteen suspected tuberculous lymphadenitis cases were evaluated in this study. Of these, 7 were excluded from the final analysis due to contamination of FNA samples during incubation. Among those of contaminated LJ tubes, discoloration of the media, liquefaction, and breaking was found in 57.1% (4/7), 28.5% (2/7), and 14.2% (1/7) tubes respectively. Of the 211 eligible TBLN suspected patients, the majority were female 61.14% (129/211) and 38.8% (82/211) were male. The mean age of the study participant was 29 years (+/- 14.45 SD) (**Table 1**). More than half of the study participants had night sweating 54.5% (115/211), poor appetite 54.5% (96/211), generalized body weakness 67.77% (143/211), whereas almost 75% (159/211) of them had no cough. 73.46% (155/211) study participants had a low rate in increase of neck Swelling. The majority of participants had weight loss 58.77% (124/211) and differences between weight loss and without individuals were found to be statistically associated with culture positivity (p = 0.012).

**Table 1:** Socio-demographic characteristics status of study participants’ data drowns from ALERT and St. Peter TB specialized hospital. Addis Ababa, Ethiopia (n = 211) 2020.

Variables		Tested on LJ (Frequency) No (%)	Culture Positive No (%)	p-value
**Age group**	0.5–10	20(9.48)	0	0.128
	11–20	43(29.86)	11(22%)	
	21–30	62(29.38)	18(36%)	
	31–40	46(21.80)	12(24%)	
	41–50	25(11.85)	7(14%)	
	>51	15(7.11)	2(4%)	
**Sex**	Male	82(38.86)	17(38%)	0.419
	Female	129(61.14)	33(66%)	
**Marital status**	Single	64(30.33)	20(40%)	0.033
	Married	84(39.81)	18(36%)	
	Divorced	7(3.32)	2(4%)	
	Widowed	17(8.06)	6(12%)	
	Living with partner	2(0.95)	1(2%)	
	Separated	4(1.90)	2(4%)	
	Living with parents	33(15.64)	1(2%)	
**Living Area**	Urban	141(66.82)	29(58%)	0.061
	Rural	70(33.17)	21(42%)	
**Occupation**	House wife	48(22.75)	11(22%)	0.142
	Daily laborer	22(10.43)	10(20%)	
	Government Employed	21(9.95)	4(8%)	
	Unemployed	42(19.91)	10(20%)	
	Farmer	21(9.95)	6(12%)	
	Other	57(27.01)	9(18%)	
**Education status**	Primary school	84(39.81)	17(34%)	0.352
	Secondary school	66(31.28)	20(40%)	
	Higher school	12(5.69)	2(4%)	
	Illiterate	43(20.38)	11(22%)	
	Other (under school age)	6(2.84)	0	

### Lymph node characteristics of study participants

Most of the lymph node aspirates were obtained from unilateral right-side 41.7% (88/211) and left-side 41.2% (87/211) lymphadenopathy. whereas the anterior and posterior cervical regions were accounted for 40.76% (86/211) and25.59% (54/211), respectively. These were followed by supraclavicular 10.9% (23/211) and axillary 9.48% (20/211). The lymphadenopathy was examined grossly for tender 22.75% (48/211) and non-tender 77.25% (163/211) lymph nodes. (**Table 2**). The number of nodes was also observed in 51.66% (109/211) for a single node of the cases, followed by few nodes (2–4 nodes) in 38.86% (82/211). Among the total cases, 66.82% (141/211) had non-mobile lymph nodes. The condition of the lymph nodes was also observed; the majority of the lymph nodes were firm 57.82% (122/211), matted 20.38% (43/211), and hard type 1.4% (3/211) of lymphadenopathy.

**Table 2:** Lymph node characteristics of study participants at ALERT and St. Peter TB Specialized Hospital. Addis Ababa, Ethiopia (n = 211) 2020.

Variables		Tested on LJ (Frequency) No (%)	Culture Positive No (%)	p-value
**Location of lymph node**				0.413
	Unilateral right sided	88(41.71%)	19(38)	
	Unilateral left sided	87(41.23%)	19(38)	
	Bilateral	35(16.59%)	12(24)	
	Generalized	1(0.47%)	0	
**Position of lymph node**				0.445
	Anterior cervical	86(40.76%)	21(42)	
	Posterior cervical	54(25.59%)	14(28)	
	Supra clavicular	23(10.90%)	6(12)	
	Axillary	20(9.48%)	7(14)	
	Mandibular	8(3.79%)	2(4)	
	Inguinal	16(7.58%)	0	
	Occipital	2(0.95%)	0	
	Femoral	1(0.47%)	0	
	Chest	1(0.47%)	0	
**Tenderness of the lymph node**				0.809
	Tender	48(22.75%)	12(24)	
	Non tender	163(77.25%)	38(76)	
**Number of nodes**				0.157
	Single node	109(51.66%)	22(44)	
	Few nodes (2–4)	82(38.86%)	20(40)	
	Multiple nodes (>5)	20(9.48%)	8(16)	
**Mobility of the nodes**				0.585
	Mobile	70(33.18%)	15(30)	
	Non mobile	141(66.82%)	35(70)	
**Condition of the nodes**				0.059
	Discrete	14(6.64%)	5(10)	
	Matted	43(20.38%)	16(32)	
	Firm	122(57.82%)	21(42)	
	Soft	5(2.37%)	0	
	Hard	3(1.42%)	0	
	Fluctuant	17(8.06%)	6(12)	
	Draining sinus	7(3.32%)	2(4)	

Cytological morphology was indicative for TB in 49.7% (105/211) of total suspected TBLN cases, of which 62.8% (66/105) were culture negatives. The cytological examination had the highest sensitivity (78%) and detection rate, but few false negatives 4.26% (9/211) were also observed. Overall, cytology had the lowest specificity 58.8%. LEDFM also had a better sensitivity of 66% and detection rate next to cytology with 77.6% specificity. Lower sensitivity (30%) was observed in ZN staining. (**Table 3**).

**Table 3:** Diagnostic accuracy of ZN, LEDFM, and cytology microscopy against culture result data drown from ALERT and St. Peter TB specialized hospital, Addis Ababa, Ethiopia. (n = 211) (2020).

ZN microscopy	LJ culture at 8th week			LEDFM	LJ culture at 8th week			Cytology	LJ culture at 8th week		
** **	**Positive**	**Negative**	**Total**		**Positive**	**Negative**	**total**		**Positive**	**Negative**	**total**
**Positive**	15	14	29	**Positive**	33	36	69	**Positive**	39	66	105
**Negative**	35	147	182	**Negative**	17	125	142	**Negative**	11	95	106
**Total**	50	161	211	**Total**	50	161	211	**Total**	50	161	211
** **	**Percentage**	**[95% CI]**			**Percentage**	**[95% CI]**			**Percentage**	**[95% CI]**	
**Sensitivity**	30%	[17.9–44.6]		**Sensitivity**	66%	[51.2–78.8]		**Sensitivity**	78%	[64–88.5]	
**Specificity**	91.3%	[85.8–95.2]		**Specificity**	77.6%	[70.4–83.8]		**Specificity**	58.8%	[50.7–66.5]	
**PPV**	35.5%	[22.2–51.4]		**PPV**	58.9%	[50.3–67.1]		**PPV**	65%	[59.4–70.1]	
**NPV**	89.1%	[87.2–90.8]		**NPV**	82.5%	[76–87.5]		**NPV**	73.1	[61.4–82.3]	
**likelihood ratio (+)**	3.45	[1.79–6.65]		**likelihood ratio (+)**	2.95	[2.08–4.19]		**likelihood ratio (+)**	1.89	[1.49–2.4]	
**likelihood ratio (-)**	0.767	[0.636–0.925]		**likelihood ratio (-)**	0.438	[0.295–0.65]		**likelihood ratio (-)**	0.374	[0.219–0.641]	

PPV = Positive predictive value. NPV = Negative predictive value.

Diagnostic efficacy of ZN microscopy, LEDFM, and Cytology was done against LJ culture. There were scanty bacilli on microscopy and a decrease in the volume of the samples was less likely to be related to a positive culture result. Of the Mycobacterial, culture-positive results were found to be 23.7% (50/211). Among those culture positives, 30% (15/50) were detected by ZN microscopy, 78% (39/50) by cytology, and 66% (33/50) by LEDFM. Among culture-negative samples, 77.6% (125/161) were correctly identified as negative by LED microscopy, 58.3% (94/161) by Cytology, and 91.3% (147/161) by ZN microscopy. The sensitivity of ZN, Cytology, and LED microscopy was 30%, 78%, and 66%, respectively against culture. The specificity of ZN microscopy was 91.3% and Cytology 58.8%, whereas 77.6% for LED microscopy. (**Table 4**).

**Table 4:** Overall diagnostic accuracy of conventional ZN microscopy, LEDFM, and cytology against culture result data drowns from ALERT and St. Peter TB specialized hospital. Addis Ababa, Ethiopia.

	ZN microscopy	LEDFM	Cytology
**Sensitivity [95% CI]**	30% [17.9–44.6]	66% [51.2–78.8]	78% [64–88.5]
**Specificity [95% CI]**	91.3% [85.8–95.2]	77.6% [70.4–83.8]	58.8% [50.7–66.5]
**NPV [95% CI]**	89.1% [87.2–90.8]	82.5% [76–87.5]	73.1 [61.4–82.3]
**PPV [95% CI]**	35.5% [22.2–51.4]	58.9% [50.3–67.1]	65 [59.4–70.1]
**Likelihood ratio (+) [95% CI]**	3.45 [1.79–6.65]	2.95 [2.08–4.19]	1.89 [1.49–2.4]
**Likelihood ratio (-) [95% CI]**	0.767 [0.936–0.925]	0.438 [0.295–0.65]	0.374 [0.219–0.641]

PPV = Positive predictive value. NPV = Negative predictive value.

There was one case detected positive by conventional ZN microscopy while reported negative by LED microscopy and cytology. Based on the cytomorphological examination, other features were reported by a pathologist as non-TB (non-specific findings) 12.8% (27/211), reactive lymphadenitis 6.6% (14/211), and malignancy 5.7% (12/211). Among the conditions with the suggestive cytomorphological examination of TBLN, 52.4% (55/105) were positive by LEDFM and 27.6% (29/105) by ZN microscopy (**Table 5**).

**Table 5:** Condition of the node and cytomorphological result comparison against LEDFM detection data drowns from ALERT and St. Peter Specialized Hospital, Addis Ababa, Ethiopia (n = 211). (2020).

LED Microscopy				
		Positive	Negative	P-value
**Condition of node**	Discrete (n = 14)	8(57.14%)	6(42.86%)	0.016
	Matted (n = 43)	18(41.86%)	25(58.14%)	
	Firm (n = 122)	33(27.05%)	89(72.95%)	
	Soft (n = 5)	1(20.00%)	4(80.00%)	
	Hard (n = 3)	0	3(100.0%)	
	Fluctuant (n = 17)	9(52.94%)	8(47.06%)	
	Draining sinus (n = 7)	0	7(100.0%)	
**Cytomorphology**	TB Lymphadenitis (n = 105)	38(36.19%)	67(63.81%)	0.0057
	Reactive lymphadenitis (n = 14)	0	14(100%)	
	Lymphoid hyperplasia (n = 6)	0	6(100%)	
	Abscess (n = 7)	3(42.86%)	4(57.14%)	
	Malignancy (n = 12)	1(8.33%)	11(91.67%)	
	Necrosis (n = 1)	0	1(100%)	
	Granulomatous lymphadenitis (n = 3)	1(33.33%)	2(66.67%)	
	Lymphoma (n = 5)	0	5(100%)	
	Anaplastic plasmacytoma (n = 1)	1(100%)	0	
	Subcutaneous lymphoma (n = 1)	0	1(100%)	
	None TB (n = 27)	3(11%)	24(89%)	
	Colloid goiter (n = 5)	0	5(100%)	
	Inconclusive (n = 7)	1(14.29%)	6(85.71%)	
	Adenoma (n = 1)	0	1(100%)	
	Giant cell tumor (n = 1)	0	1(100%)	
	Inflamed keratin cyst (n = 1)	0	1(100%)	
	None specific adenitis (n = 3)	0	3 (100%)	
	None specific lymphadenitis (n = 1)	0	1(100%)	
	Small mature lymphoma (n = 1)	0	1(100%)	
	Sub-cutaneous lymphoma (n = 1)	0	1(100%)	
	Other (n = 8)	2(25%)	6(75%)	

## Discussion

The low bacterial load in the specimen makes routine laboratory procedures challenging to diagnose TB lymphadenitis. In these cases, invasive examinations might be necessary like FNA and Biopsy. However, the FNA sample has different sensitivity for the diagnosis of LNTB in various methods. The sensitivity of cytological examination is the highest especially in the FNA sample compared to other methods [11].

WHO recommends the replacement of conventional ZN microscope by LED microscope, and that LED microscopy be staged as an option for conventional Ziehl-Neelsen microscopy [23]. These days, studies have investigated the performance of LED microscopy for the direct detection of TB lymphadenitis especially in high incidence countries like Ethiopia. In this study, there were statistically significant differences in sensitivity between LEDFM and ZN microscope with a good overall agreement of 66% [52.9–79.7] 95% CI (**Table 4**). This is a significant finding and supports previous research that showed fluorescence microscopy has better diagnostic efficacy than conventional ZN microscopy [6, 12].

The lower specificity of LED microscopy contrasted with conventional ZN microscopy has been described before [12, 18, 24]. In our study, the specificity of LED microscopy was marginally lower than that of ZN microscopy. However, the observed difference was not noteworthy. Overall, in this study, we found that the result of scanty bacilli under the microscope and a decrease in the collected FNA volume were less likely to be related to the yield of culture positivity. Mycobacteria from these paucibacillary samples might have been killed during the FNA sample processing procedure and fail to grow in culture [9, 12].

Time-saving with LEDFM can be attributed to prompt filtering of each field because of improved detection of Mycobacteria. Slides can be analyzed at lower 40x magnifications, consequently permitting the assessment of extensive field for per unit of time. The decreased magnification utilized with LEDFM compared with light microscopy may have contributed towards the slight affectability contrasts noted as has as of now been referenced in different investigations [25–27]. The presentation of driven LEDFM in a high TBLN incidence setting would hence fundamentally minimize lab workload and perhaps permit better quality microscopy to be cultivated with a similar human resource contrasted with ZN microscopy [25].

In terms of sensitivity and specificity LEDFM and cytological examination demonstrate different advantages over each other [12]. This might be due to in LED microscopy, TB bacilli stood apart as splendid particles against a black background of the microscope, which makes them effectively recognizable and thus causing less eye strain and quick evaluation of the bacillus. Cytology has generally high sensitivity in any case with lower specificity in overall agreement [12, 16, 24, 25, 28, 29]. As the cytological examination is less specific and the ZN microscope is less sensitive as studied by this and previous studies [12, 25, 28] it is recommended to supplement the demonstrative algorithm with LED microscopy within the existing and future laboratories. Also, especially in the rural areas of the countries where FNA cytology laboratories are unavailable, LED-based microscopy can be an option. Taking everything into account, our investigation affirms that LED microscopy has higher sensitivity and a bit lower specificity in examining TB bacilli in the FNA sample when contrasted with ZN microscopy. The use of cytology in combination with LED microscopy can enhance specificity and also improves the proper identification or diagnosis of patients with TBLN.

Furthermore, the more drawn-out life expectancy of the LED frameworks, the quicker reading time of smears, and convenience would make this instrument best for fringe diagnostic facilities in areas with limited resources to improve proof-based finding and treatment of TBLN.

## Conclusion and recommendations

In conclusion, our research reveals that the LED fluorescence microscopy gives a legitimate option in contrast to conventional ZN techniques in terms of its higher sensitivity, and a bit lower specificity, time-saving and minimal effort for the diagnosis of TBLN. In resource-constrained environments, LED-based fluorescent microscopy is the most realistic choice for improved TBLN case finding. To evaluate the cost-effectiveness and feasibility of using some field analysis is recommended. Additionally, the conventional ZN microscopy can be replaced with LED-based fluorescence microscopy and incorporated in the current algorithm as a first-line laboratory test for the diagnosis of tuberculous lymphadenitis.

## Supporting information

S1 File Interpretation of the tests. (DOCX)
