# Supplementary material for: Diagnostic efficacy of Light-Emitting Diode (LED) Fluorescence based Microscope for the diagnosis of Tuberculous lymphadenitis
Source: PLoS One. 2021 Jul 29;16(7):e0255146. doi: 10.1371/journal.pone.0255146 (PMC8320901; doi:10.1371/journal.pone.0255146)
Supplement: S1 File — (DOCX) [file pone.0255146.s001.docx]

**S1 File**

***Table 1. Interpretation of LEDFM Quantification (Global Laboratory initiatives)***

| IUATLD/WHO scale (1000x field = HPF) Result | Fluorescence Microscopy Quantification System |  |
| --- | --- | --- |
|  | (200–250x magnification: 1 length = 30 fields = 300 HPF) | (400x magnification: 1 length = 40 fields = 200 HPF) |
| Negative | Zero AFB / 1 length | Zero AFB / 1 length |
| Scanty | 1–29 AFB / 1 length | 1–19 AFB / 1 length |
| 1+ | 30–299 AFB / 1 length | 20–199 AFB / 1 length |
| 2+ | 10–100 AFB / 1 field on average | 5–50 AFB / 1 field on average |
| 3+ | >100 AFB / 1 field on average | >50 AFB / 1 field on average |

***Table 2. Interpretation of Bright-field Microscope Quantification (global laboratory initiatives)***

| IUATLD/WHO scale (1000x field = HPF) Result | Bright-field Microscopy Quantification system |
| --- | --- |
|  | Bright-field (1000x magnification:1 length = 2 cm = 100 HPF) |
| Negative | Zero AFB / 1 length |
| Scanty | 1–9 AFB / 1 length or 100 HPF |
| 1+ | 10–99 AFB / 1 length or 100 HPF |
| 2+ | 1–10 AFB / 1 HPF on average |
| 3+ | >10 AFB / 1 HPF on average |

***Table 3. Interpretation of LJ Culture Quantification (World health organization)***

| Growth | Laboratory report | ZN result | Study report |
| --- | --- | --- | --- |
| None | No growth | NA | Negative for MTB complex |
| 1-9 colonies | Record Actual number | Positive | TB growth (1-9 colonies). |
| 10-100 colonies | 1+ | positive | TB growth (10-100 colonies) |
| >100-200 colonies | 2+ | Positive | TB growth (more than 100 colonies). |
| >200 colonies (too numerous to count) | 3+ | Positive | TB growth (innumerable or confluent). |
| Other Mycobacterial growth | Positive for other Mycobacteria | Positive | No MTB complex growth, but positive for other Mycobacteria. |
| Contamination | Contaminated | NA | Contaminated |
| ZN positive in presence of contamination | Positive for MTB and Contamination | Positive | Positive for MTB complex and contamination |
